# Electrodeposition as an Alternative Approach for Monolithic Integration of InSb on Silicon

**DOI:** 10.3389/fchem.2021.810256

**Published:** 2022-01-20

**Authors:** Katarzyna E. Hnida-Gut, Marilyne Sousa, Marinus Hopstaken, Steffen Reidt, Kirsten Moselund, Heinz Schmid

**Affiliations:** ^1^ IBM Research Europe-Zurich, Rüschlikon, Switzerland; ^2^ IBM T.J. Watson Research Center-Yorktown Heights, New York, NY, United States

**Keywords:** integration, InSb, electrodeposition, recrystallization, III-Vs, TASE

## Abstract

High-performance electronics would greatly benefit from a versatile III-V integration process on silicon. Unfortunately, integration using hetero epitaxy is hampered by polarity, lattice, and thermal expansion mismatch. This work proposes an alternative concept of III-V integration combining advantages of pulse electrodeposition, template-assisted selective epitaxy, and recrystallization from a melt. Efficient electrodeposition of nano-crystalline and stochiometric InSb in planar templates on Si (001) is achieved. The InSb deposits are analysed by high resolution scanning transmission electron microscopy (HR-STEM) and energy-dispersive X-ray spectroscopy (EDX) before and after melting and recrystallization. The results show that InSb can crystallise epitaxially on Si with the formation of stacking faults. Furthermore, X-ray photoelectron (XPS) and Auger electron (AE) spectroscopy analysis indicate that the InSb crystal size is limited by the impurity concentration resulting from the electrodeposition process.

## Introduction

Globally, one of the fastest-growing industries is electronic devices manufacturing based on silicon technology. With an increasing demand for faster, smaller, and better-performing devices, growth CAGR (compound annual growth rate) of 4.6% from 2020 to 2027 for this field is expected. Nonetheless, recently both academia and industry have focused efforts on the search for alternative technologies for which performance in the long run could considerably surpass that of Si (Ramirez et al., 2020), especially in more futuristic applications like quantum computing. These include III-V semiconductors for sensing and high-speed electronics where high value is created (Riel, 2017). The best solution would be to combine the advantages of Si and III-Vs (Hopkinson et al., 2013). However both economic and technological difficulties, like crystal lattice and polarity mismatch, have prevented the integration of foreign materials directly on a silicon platform.

One approach to address this problem, the so-called Template-Assisted Selective Epitaxy (TASE), was developed by IBM a few years ago (Schmid et al., 2015). By directing the growth by an oxide template and using metal-organic vapor phase deposition (MOCVD) to fill the designed structures, successful integration of InGaAs, InP, GaSb, etc. for high-performance MOSFETs or lasers on silicon was possible. However, MOCVD growth is limited by its low growth rates, difficulty of filling high aspect ratio structures, and the use of toxic precursors. Furthermore, not every III-V semiconductor is well suited for this technique. Examples for challenging materials include aluminum containing compounds due to the high sticking coefficient of Al on the mask material and antimonides due to the small process window to supress droplet formation. InSb is a particularly interesting material with properties such as one of the smallest direct band gap (0.17 eV at 300 K) and highest electron mobility (77,000 cm^2^V^−1^s^−1^) among III-Vs and can ensure applications in a wide range of modern devices, starting from terahertz (Liu et al., 2018a) and gamma rays detectors (Hishiki et al., 2005), through IR (Xie et al., 2016; Shi et al., 2019) and neutron-resistant Hall (Jankowski et al., 2019) sensors, ending on topological quantum devices for Majorana investigation (Chen et al., 2021). As an alternative synthesis path, electrochemical technique is considered since it was demonstrated to fill different shapes and sizes of templates efficiently, with control over composition, and using a simple set-up with water-based electrolytes (Hnida et al., 2015; Hnida et al., 2019). However, due to the characteristics of the InSb electrodeposition, the obtained material is polycrystalline, which significantly limits its practical use. To overcome this, concepts borrowed from micro zone recrystallization (Billings, 1969) and rapid melt growth (Liu et al., 2018b) can be applied. Here, the samples are subjected to a short high temperature step exceeding the melting temperature. During this step the polycrystalline sample melts and recrystallizes from a seed interface, resulting in a single crystalline structure with epitaxial relationship to the substrate.

**FIGURE 1:**
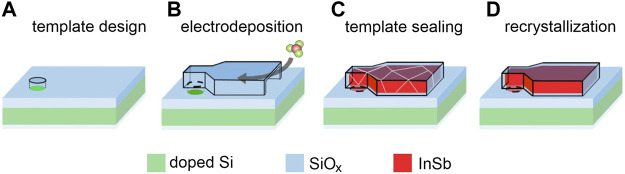
Proposed process flow for InSb integration on Si. Conductive Si substrate with surrounding SiO_x_ insulation **(A)** Conductive Si substrate with surrounding SiOx insulation and **(B)** 3D template is immersed in the electrochemical cell. **(C)** After electrochemical deposition of polycrystalline InSb, the template is sealed. **(D)** Melting and recrystallization to form a InSb singe crystal on Si.

The goal of this work is to directly integrate InSb on a silicon platform. To reach that we propose combining the three techniques, namely TASE, pulse electrodeposition, and recrystallization. The proposed process is designed to resolve the identified issues and is summarized in Figure 1. One of the key aspects is that the low melting point of InSb could allow for a CMOS compatible process.

## Materials and Methods

One of the requirements for electrodeposition of InSb on silicon is to provide a conductive electrode. In the presented work we used highly doped silicon wafer as global working electrode to simplify the fabrication process, while formation of addressable electrodes would require additional processing steps. Silicon wafers (001) n-type for two sets of templates having larger or smaller structures were prepared. For template fabrication, holes were etched in 100 nm thick SiO_x_ on Si by reactive ion etching (RIE) to define the electrode/seed area. Then, a 1,350 nm thick bis-benzocyclobutene (BCB, Dow) layer was spin coated and cross-linked at 320°C. Subsequently, the BCB layer was patterned with the layout of template structures, etched using RIE, and coated with a 150 nm thick SiO_x_ shell by plasma enhanced chemical vapor deposition (PECVD). Finally, openings were patterned on the template structures and etched into the SiO_x_ shell exposing the BCB layer. The remaining BCB layer was etched using peroxide/sulfuric acid 1:3 solution, resulting in hollow template structures. The as-prepared substrate was dipped for 12–15 s in diluted (2.5%) HF to remove the native oxide on the exposed Si surfaces within the channels before electrodeposition.

**FIGURE 2:**
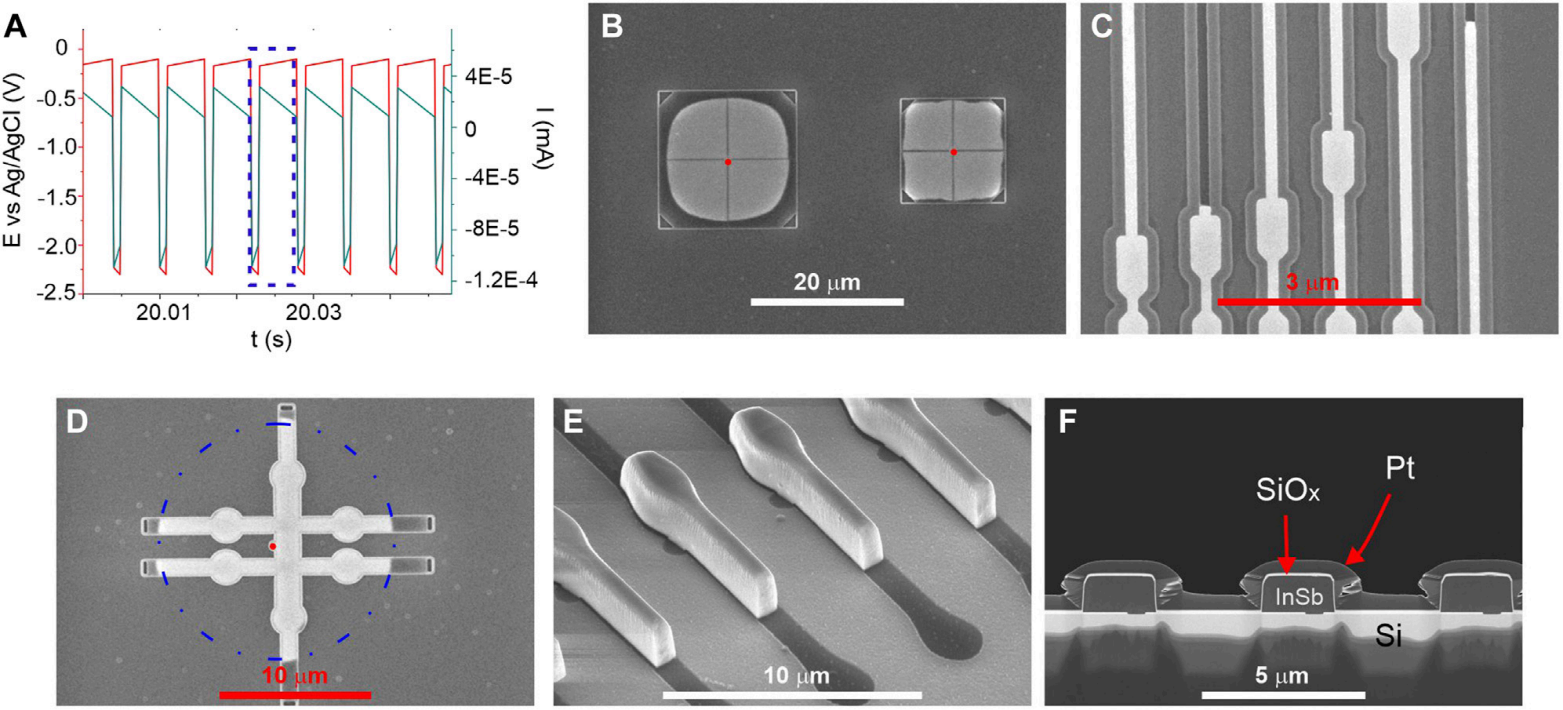
**(A)** Section of typical potential pulse sequence. Blue dotted frame marks one cycle. **(B–D)** SEM images of filled SiO_x_ templates with different shapes and sizes. Seed hole marked with the red dot. **(E)** SEM image of InSb deposit after partial removal of the SiO_x_ template. The dark shade marks the area of the template which was left unfilled. **(F)** A cross-section of filled templates exposing the seed regions. Both SiO_x_ capping (thin bright part on top of the deposit) and Pt proctective (thick grey part on top of the SiO_x_) layers are marked.

All electrochemical syntheses were performed in a water-based electrolyte containing 0.06M InCl_3_ (Sigma Aldrich, >98%), 0.045M SbCl_3_ (Sigma Aldrich, ≥99%), 0.2M citric acid (Sigma Aldrich, ≥99%), and 0.17M sodium citrate (Sigma Aldrich, ≥99%) at room temperature. Both citric acid and sodium citrate were used as complexing agents to bring reduction potentials of indium and antimony ions closer to each other. The electrodeposition experiments were carried out using the potentiostat (BioLogic SP300) with a three-electrode setup. An Ag/AgCl (3M NaCl) electrode as a reference electrode (Ref) and Pt wire as a counter electrode (CE) were employed. SiO_x_ template as a working electrode (WE) was installed at a distance of 1.5 cm from the CE electrode. Unless otherwise stated, all potentials refer to the Ref. The fabrication of InSb nano and microstructures was performed using a cathodic pulse with E_on_ = –2.3 V followed by E_off_ = −0.1 V. The potentials of “on” and “off” pulses were chosen based on Linear Sweep Voltammetry (LSV) and Open Circuit Potential (OCP) measurements on bare n-Si, respectively. The duration of pulse “on” and “off” were 1 and 5 ms, respectively, based on previous research (Hnida et al., 2015). During pulse “on” the reduction of In and Sb citric complexes occur, while during pulse “off” replenishment of electrolyte near the working electrode surface and dissolution of the top surface of deposit happen (Rajska et al., 2021). Section of the typical electrodeposition pulse sequence is presented in Figure 2A. Blue dotted frame marks one cycle. Slight variations in potential and current (red and green lines, respectively, slope especially visible for pulse “off”) are related to the data accusation. The total deposition times ((t_on_ + t_off_) × number of cycles) were kept in a range of 12–240 s depending on the size and shape of the oxide template and overall geometrical area of the working electrode.

For a thin intermediate InSb layer synthesis, MOCVD technique was implemented. Selective epitaxy of InSb was carried out using trimethylindium (TMIn), and trimethylantimony (TMSb) at V/III ratio 100 at 430°C for 65 s, followed by 25 s of Sb deposition to further suppress the presence of In droplets.

The melting process was performed on SiO_x_-capped samples at 535°C, 50 mbar for 2 s. The system was calibrated prior to the process and offset in the measured temperature was considered. After annealing, the SiO_x_ capping layer was removed using a combination of RIE and HF etching (either wet or vapor), and the morphology, crystallinity, and chemical composition were characterized using scanning electron microscopy (SEM, Hitachi SU8000) and analytical (scanning) transmission electron microscope ((S)TEM, JEOL ARM200F) equipped with energy-dispersive X-ray spectroscopy detector. Lamellas for (S)TEM-EDX analysis were prepared using the focus ion beam technique (Dual beam FIB/SEM, FEI Helios NanoLab 450S). Priori the lamella preparation samples were covered with 250 nm of SiO_x_. The samples have been measured under UHV condition with a Varian Auger system equipped with a TPIS plasma gun.

## Results and Discussion

In preliminary studies, we tested different silicon wafer orientations and doping levels. No change in morphology and composition between deposits was observed as long as ohmic contacts could be established. InSb deposition on BHF passivated Si showed a high nucleation density and dense film formation. However, the film adhesion was low and with the buildup of mechanical strain in the film, leading to delamination with increasing thickness already in the plating solution (Hnida-Gut et al., 2021). Figure 2 shows representative SEM images of InSb deposited in the template structures. Here, loss of adhesion and strain as encountered with planer films is not a limiting factor anymore. Figure 2B shows two squares composed of four quadrants each with the seed located in the center, and the template opening at the corners. The deposition process is very selective, and the InSb is deposited with high uniformity in each quarter. Templates with up to 10 µm large free-standing structures were successfully filled, while larger ones collapsed due to capillary forces during the fabrication process. The smallest structures fabricated and filled (Figure 2C) have line width down to 120 nm. Figure 2D illustrates uniform filling of the intended Hall bar structure with the seed area indicated as red point and a dashed circle as reference. Figure 2E shows a tilted view image of a sample where the SiO_x_ was partially removed. The smooth top surface is a result of the replication of the inherently low surface roughness of the spin coated BCB film, while the sidewall roughness originates from the BCB etching process. Cross-sections from several structures are shown in Figure 2F, confirming the absence of gaps, voids, and cracks in the InSb.

**FIGURE 3:**
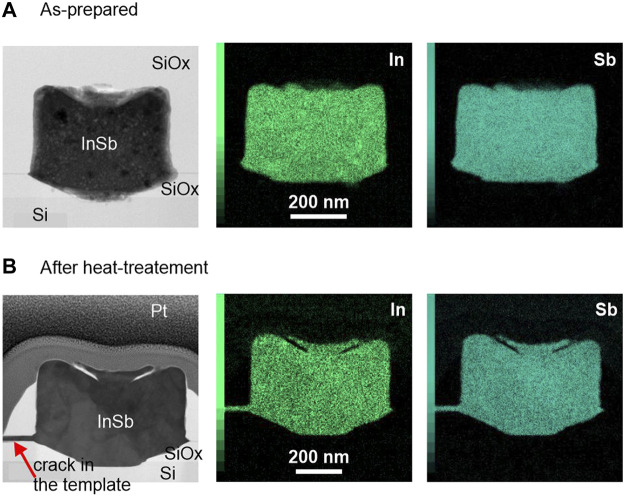
(S)TEM images and EDX maps for **(A)** as-prepared and **(B)** annealed InSb deposits. The scale bar for all images is 200 nm.

After demonstrating the successful electrodeposition on InSb on Si, and according to Figure 1, step D follows with melting and controlled recrystallization of the InSb from the seed area. First, the chemical composition is accessed before and after the melting step, followed by further chemical and structural analysis. Figure 3 shows (S)TEM cross-section images of two samples (as-prepared and annealed, respectively) which were cut across the samples such as to expose seed areas and including the InSb-Si interface. We note that the unexpected roughness of the top surfaces stems from an artefact during the fabrication process. (S)TEM-EDX mapping was performed to investigate the distribution of In and Sb elements (Figure 3, center and right-side panels) in the deposited volume before and after annealing for 2 s at 535°C. In both samples the distribution of In and Sb atoms is uniform with no detectable phase separation. The In/Sb ratio for the as-prepared sample was 45/55 (±10%) and remained constant after annealing. In Figures 3A,B a small crack is visible on the left (marked with the red arrow) which was created during the annealing procedure, caused by volume expansion of the melt or thermal stress in the oxide template. Interestingly, the resulting void is completely filled with InSb, thus confirm the melting of the deposit. After the RTA process voids in the InSb structures were frequently detected by SEM inspection, indicating loss of material. This observation was also reported in Menon et al. (2021) and needs further investigation.

**FIGURE 4:**
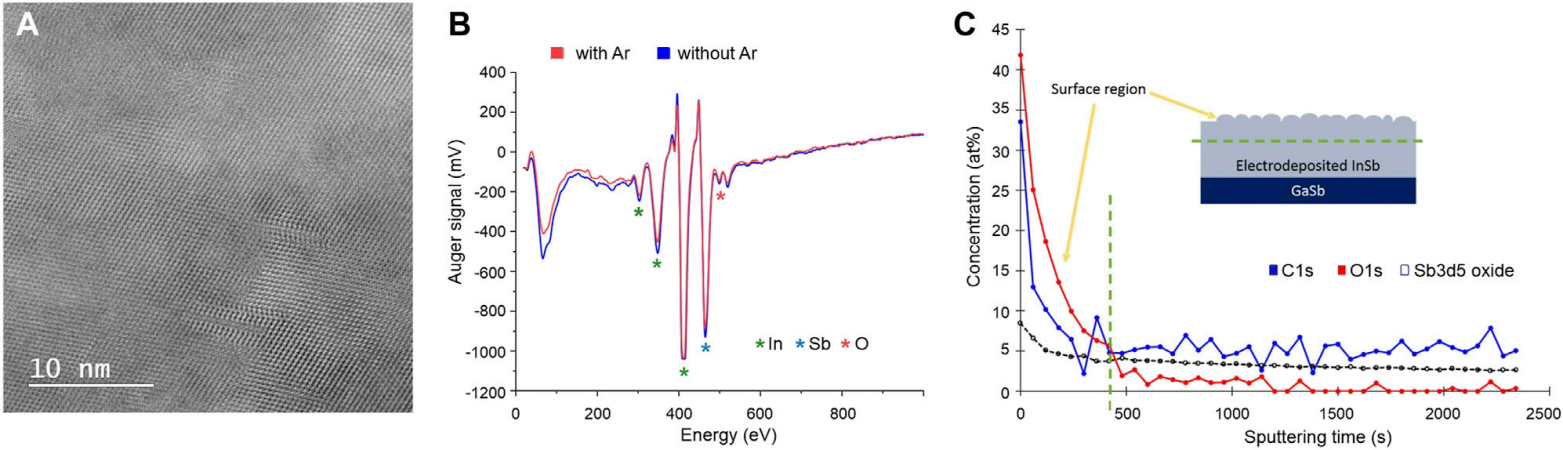
**(A)** High-resolution (S)TEM image of InSb after annealing process. **(B)** Auger spectra for samples electrodeposited with (blue) and without (red) oxygen dissolved in the electrolyte, respectively. **(C)** XPS depth profile of impurities [O (red), C (blue), and Sb oxide (black)] concentration (at%) distribution in electrodeposited InSb. Sputtering time is directly correlated with the thickness of the deposit. A green dotted line marks the surface region with indicates the presence of electrolyte residues and surface oxides.

Structural analysis using bright field (BF) HR-(S)TEM revealed unexpectedly that both samples remained throughout nanocrystalline with no significant differences in the size of the crystals (Figure 4A), despite the fact that the melting was indeed achieved. In addition, the anticipated recrystallization from the Si seed interface did not take place. Regarding the first observation, a possible explanation might be found in the process of diamond crystallization in metal-carbon systems. Palyanov et al. recently reported that oxygen can greatly affect the crystallization (Palyanov et al., 2020). They found that the concentration of O_2_ higher than 0.25 wt% can lead to spontaneous nucleation events which hinders crystal growth. As oxygen is present in the water-based electrolyte used for InSb electrodeposition, it is plausible that impurity induced nucleation is limiting the InSb crystallization. The common way of removing oxygen dissolved in the electrolyte is to deoxygenate it with inert gas (such as N_2_ or Ar) before and during the synthesis. To explore this hypothesis, two electrodeposition processes were conducted (namely with and without O_2_ dissolved in the electrolyte), after which samples were immediately stored in a glove box. Both samples were subjected to Auger electron spectroscopy measurement to determine the influence of O_2_ in the electrolyte on its content in the InSb deposits. Both spectra are presented in Figure 4B and reveal that regardless of whether the electrolyte used in the synthesis was deoxygenated or not, significant amounts of oxygen are incorporated. To further confirm the intrinsic oxygen and/or carbon contamination in electrodeposited InSb, XPS analysis was performed using a GaSb as substrate for Sb-signal as reference. Due to overlapping spectra for Sb and O, the exact deduction of how much oxygen is dissolved in the InSb deposit is, however, challenging. The XPS depth profile is shown in Figure 4C and confirms high concentrations of oxygen, carbon, and antimony oxide on the surface and in the bulk of the electrodeposited InSb. Impurity concentrations are stable around 5.2 at%, 0.8 at%, and 3.1 at% for C, O, and Sb oxide, respectively, which can explain the observed suppression of crystal growth.

**FIGURE 5:**
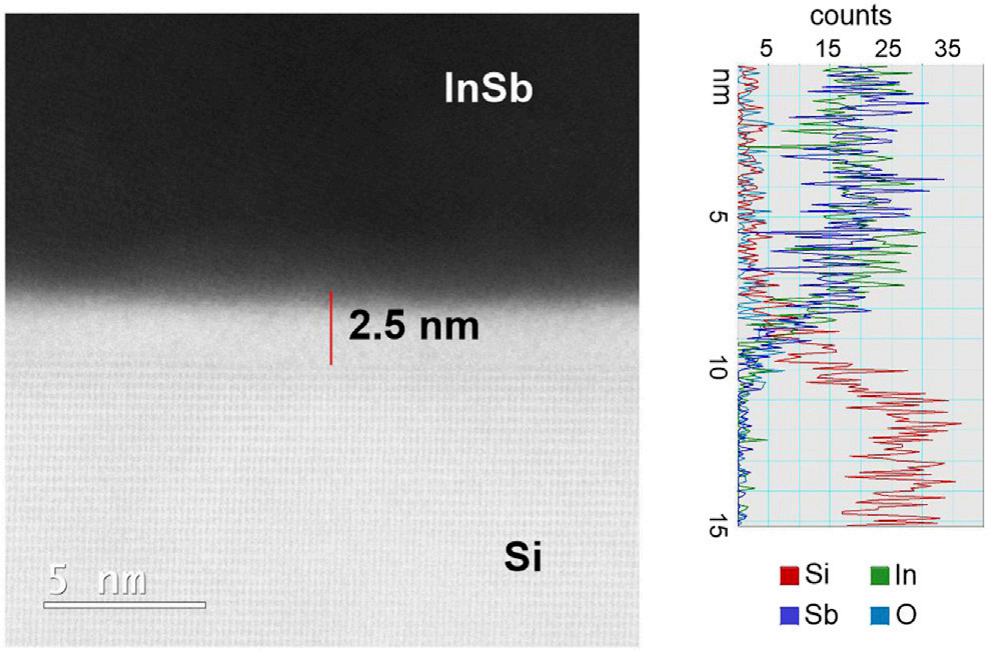
High-resolution (S)TEM image of the Si—InSb interface for recrystallized sample together with EDX spectra.

As noted, the anticipated recrystallization from the Si interface is not observed, and in addition it is expected that the liquid melt should lead to a dissolution and therefore local etching of the Si surface which is also not observed in Figure 3B. A BF HR-(S)TEM of the interface of the annealed sample is presented in Figure 5 and reveals a 2.5 nm thick amorphous (barrier) layer between Si seed (bottom bright part) and InSb deposit (dark part), whose presence inhibits epitaxial contact between the Si and the InSb melt and correspondingly also etching of the Si surface. The composition of the barrier was further evaluated using EDX, and together with the (S)TEM data indicates the presence of Si- and metal (In, Sb) oxides. The unhindered effectiveness of the electroplating suggests that the amorpous barrier layer acts mainly as material diffusion barrier and barrier for epitaxy, but not as effective electrical insulator. Despite its 2.5 nm thickness, direct tunneling, and more importantly trap assisted tunneling (TAT) can be significant at ∼1 Vnm^−1^ in low density oxides. In fact, electron tuneling at high cathodic voltages was previously observed during Cu electrodeposition on thin native silicon oxide (Arrington et al., 2008). Moreover, the barrier may contain intrinsically conductive In or Sb oxides. To overcome the formation of a barrier layer, which might itself be intrinsic to the specific electrodeposition process, a modified strategy is demonstrated in the following section.

**FIGURE 6:**
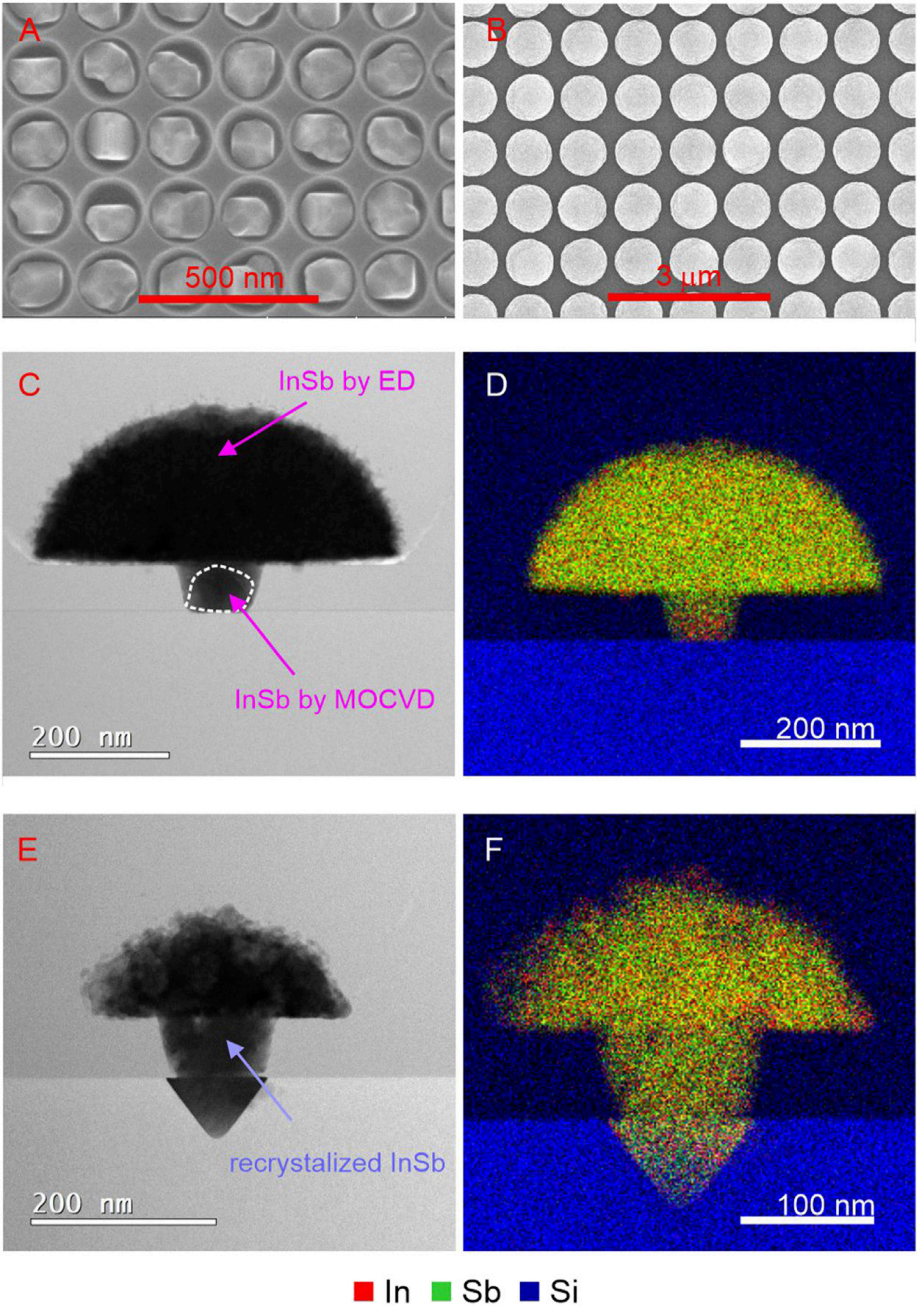
**(A)** SEM images of dense array of InSb crystallites by MOCVD serving as seeds for later electrodeposition. **(B)** sparser array similar to **(A)** with the small InSb seeds after the electrodeposition step resulting in large InSb mushrooms. **(C)** (S)TEM cross-section image of a representative structure from **(B)** together with **(D)** EDX map of In, Sb, and Si elemental distribution. **(E)** (S)TEM image of **(B)** after melting and crystallization. **(F)** EDX map of **(E)**.

To demonstrate the overall validility of the concept illustrated in Figure 1, the formation of a barrier layer during electrodeposition must be excluded. This small modification is introduced by selectively depositing a thin intermediate layer of InSb using MOCVD in the Si seed areas. This guarantees an oxide free InSb-Si interface which serves in a later step as interface for the electrodeposition of InSb, and the subsequent steps demonstrated above. Instead of complex templates, arrays of vias in a SiO_x_ mask were patterned on Si (001) and used as a mask for MOCVD growth of small InSb crystals. The conditions of epitaxial growth were chosen to minimize the presence of In droplets on the InSb crystals, with randomly occurring In droplet still detectable (for detailed description please refer to section 2 Materials and Methods). The resulting InSb seeds crystals are shown in Figure 6A. Each hole contains at least one InSb crystal protecting the InSb-Si interface from reoxidation processes. Directly after MOCVD synthesis, the electrodeposition process was performed in which each 100 nm-sized InSb crystallite was further overgrown by InSb, resulting in >500 nm sized domes (Figure 6B). Finally, the samples were capped with SiO_x_. The results of (S)TEM-EDX analysis of as-prepared and melted/crystallized samples are presented in Figures 6C–F. The arrows in Figure 6C point to the crystalline InSb deposited by MOCVD (for better visibility marked with white dotted line) and to the nanocrystalline InSb deposited by ED. The surface roughness of the ED process at is clearly visible around the perimeter of the mushroom. The corresponding EDX map in Figure 6D confirms a uniform composition of the ED part, but also a slight In rich area in the InSb seed which might be caused by a In droplet formed during MOCVD growth. The differences of intensities are simply a consequence of the variable lamella thickness, with the stem being thinner compared to the hemisphere. As expected InSb-Si interface is flat.

After melting and crystallization, the morphology changes significantly, as visible in Figure 6F. According to the phase diagram, the melted phase gets saturated with Si, leading to a dissolution of Si, confirmed by the presence of the inverted pyramidal structure bound by four Si <111> planes below the seed region. This melt-back etching is a well documented phenomenon and is investigated in detail for gallium (Werner et al., 2014) and gallium nitride (Khoury et al., 2017) epitaxial growth on Si, for example. Upon cooling, the dissolved Si precipitates out again until reaching the equilibrium solubility limit. At first surprising is the large volume of Si that is melt-back etched, corresponding to approximately 15% of the entire InSb volume, by several orders exceeding the solid solubility limit of Si in In (Olesinski et al., 1985) and Sb (Olesinski and Abbaschian, 1985). A closer look at the structure after melting and crystallisation reveals a much increased surface roughness along the perimeter and a correspondingly decreased radius of the hemisphere. This suggests that dissolved Si was diffusing through the InSb melt and precipitating out again on the opposing interface during the melt phase. The etching process slows down when the stable <111> planes are formed, defined by the mask opening, but still continues at reduced speed as is noticeable by the underetching of the mask pattern. EDX measurements (Figure 6F) could not confirm the hypothesis by revealing a distinct Si signal at a periphery, due to the overlap of the Si signal stemming from the SiO_x_ capping layer, and due to the likelihood of the Si forming a SiO_x_ compound as well considering the high O level in the InSb.

**FIGURE 7:**
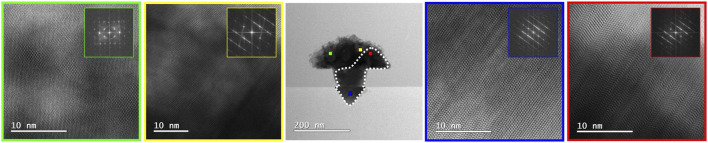
(S)TEM analysis of the melted and crystallized InSb structure. The region with an epitaxial relationship to the substrate is marked with the white dotted line. BF HR-(S)TEM images marked with red and blue show the zinc blend monocrystalline region of the InSb structure. The remaining part of the structure is nano crystalline and marked with yellow and green.

Finally, structural analysis of the crystallized sample is shown in Figure 7, where an extended monocrystalline area was found (delineated by a dashed line) with the rest remaining nano crystalline. BF HR-(S)TEM revealed that this single crystalline area has the same epitaxial relationship as the Si (001) substrate, confirming the anticipated process of initial crystallization of the melt from the Si interface. The single crystal has a high density of the stacking faults which run parallel to one of the four possible <111> planes of the substrate. This is the first demonstration that the proposed concept is indeed feasible, although on a limited scale only due to the limit dictated by the impurity concentration of the InSb melt. It is worth noting that using a higher melting point semiconductor such as InAs in place of the InSb crystal deposited by MOCVD in this work, melt-back etching of the Si and therefore unintentional Si doping of the InSb can be avoided.

## Conclusion

We have proposed and demonstrated the use of electrodeposition as an alternative approach for monolithic integration of a compound semiconductor on silicon. Arbritrary shaped templates were efficiently filled with nanocrystalline InSb from an aqueous solution using pulsed electrodeposition. The melted and crystallized InSb within the templates were found to remain nanocrystalline, which was ascribed to a high impurity concentration in the InSb. Complexing additives to match the reduction potentials of indium and antimony ions closer together are proposed as a source of the impurities and heterogeneous nucleation during melt cooling. Despite the limitation encountered, InSb plating and epitaxial melt crystallization from a Si surface was demonstrated on a sub-micron scale, validating the proposed integration concept. To improve the current results towards large area epitaxial InSb films on Si, a synthesis path including brightener additives to form high purity deposits will be explored.

## Data Availability

The raw data supporting the conclusion of this article will be made available by the authors, without undue reservation.

## References

[B1] Arrington D. Curry M. Street S. Pattanaik G. Zangari G. (2008). Copper Electrodeposition onto the Dendrimer-Modified Native Oxide of Silicon Substrates. Electrochimica Acta 53, 2644–2649. 10.1016/j.electacta.2007.10.054

[B2] Billings A. R. (1969). Microzone Recrystallization of Semiconductor Compound Films. J. Vacuum Sci. Tech. 6, 757–765. 10.1116/1.1315753

[B3] Chen Y. Huang S. Pan D. Xue J. Zhang L. Zhao J. (2021). Strong and Tunable Spin-Orbit Interaction in a Single Crystalline InSb Nanosheet. Npj 2d Mater. Appl. 5, 3. 10.1038/s41699-020-00184-y

[B4] Hishiki S. Kanno I. Sugiura O. Xiang R. Nakamura T. Katagiri M. (2005). Undoped InSb Schottky Detector for Gamma-ray Measurements. IEEE Trans. Nucl. Sci. 52, 3172–3175. 10.1109/TNS.2006.869817

[B5] Hnida-Gut K. E. Sousa M. Moselund K. E. Schmid H. (2021). “Direct Electrodeposition of InSb Devices on Silicon,” in Conference Proceedings of 16th IEEE Nanotechnology Materials and Devices Conference (IEEE NMDC 2021), Vancouver, Canada, 12–15.12.2021. in press.

[B6] Hnida K. E. Bäβler S. Akinsinde L. Gooth J. Nielsch K. Socha R. P. (2015). Tuning the Polarity of Charge Transport in InSb Nanowires via Heat Treatment. Nanotechnology 26, 285701. 10.1088/0957-4484/26/28/285701 26112309

[B7] Hnida K. E. Marzec M. Wlaźlak E. Chlebda D. Szaciłowski K. Gilek D. (2019). Influence of Pulse Frequency on Physicochemical Properties of InSb Films Obtained via Electrodeposition. Electrochimica Acta 304, 396–404. 10.1016/j.electacta.2019.02.111

[B8] Hopkinson M. Martin T. Smowton P. (2013). III-V Semiconductor Devices Integrated with Silicon. Semicond. Sci. Technol. 28, 090301. 10.1088/0268-1242/28/9/090301

[B9] Jankowski J. Prokopowicz R. Pytel K. El-Ahmar S. (2019). Toward the Development of an InSb-Based Neutron-Resistant Hall Sensor. IEEE Trans. Nucl. Sci. 66, 926–931. 10.1109/TNS.2019.2912720

[B10] Khoury M. Tottereau O. Feuillet G. Vennéguès P. Zúñiga-Pérez J. (2017). Evolution and Prevention of Meltback Etching: Case Study of Semipolar GaN Growth on Patterned Silicon Substrates. J. Appl. Phys. 122, 105108. 10.1063/1.5001914

[B11] Liu Y. Kanyang R. Han G. Fang C. Zhang J. Hao Y. (2018a). “Rainbow Trapping and Releasing in InSb Graded Grating Strip at the Terahert: Range,” in 2018 Cross Strait Quad-Regional Radio Science and Wireless Technology Conference (CSQRWC), Xuzhou, China, 21–24.07.2018 (IEEE), 1–3. 10.1109/CSQRWC.2018.8455539

[B12] Liu Z. Wen J. Li C. Xue C. Cheng B. (2018b). Research Progress of Ge on Insulator Grown by Rapid Melting Growth. J. Semicond. 39, 061005. 10.1088/1674-4926/39/6/061005

[B13] Menon H. Södergren L. Athle R. Johansson J. Steer M. Thayne I. (2021). Improved Quality of InSb-On-Insulator Microstructures by Flash Annealing into Melt. Nanotechnology 32, 165602. 10.1088/1361-6528/abd656 33361572

[B14] Olesinski R. W. Abbaschian G. J. (1985). The Sb-Si (Antimony-Silicon) System. Bull. Alloy Phase Diagrams 6, 445–448. 10.1007/BF02869508

[B15] Olesinski R. W. Kanani N. Abbaschian G. J. (1985). The In−Si (Indium-Silicon) System. Bull. Alloy Phase Diagrams 6, 128–130. 10.1007/BF02869223

[B16] Palyanov Y. N. Borzdov Y. M. Kupriyanov I. N. Bataleva Y. V. Nechaev D. V. (2020). Effect of Oxygen on Diamond Crystallization in Metal-Carbon Systems. ACS Omega 5, 18376–18383. 10.1021/acsomega.0c02130 32743213 PMC7391949

[B17] Rajska D. Brzózka A. Hnida-Gut K. E. Sulka G. D. (2021). Investigation of Electrodeposition Kinetics of in, Sb, and Zn for Advanced Designing of InSb and ZnSb Thin Films. J. Electroanalytical Chem. 882, 114967. 10.1016/j.jelechem.2020.114967

[B18] Ramirez J. M. Malhouitre S. Gradkowski K. Morrissey P. E. O'Brien P. Caillaud C. (2020). III-V-on-Silicon Integration: From Hybrid Devices to Heterogeneous Photonic Integrated Circuits. IEEE J. Select. Top. Quan. Electron. 26, 1–13. 10.1109/JSTQE.2019.2939503

[B19] Riel H. (2017). “Integrated III-V Nanoelectronic Devices on Si,” in 2017 Silicon Nanoelectronics Workshop (SNW), Kyoto, Japan, 4–5.06.2017 (IEEE), 1–2. 10.23919/SNW.2017.8242267

[B20] Schmid H. Borg M. Moselund K. Gignac L. Breslin C. M. Bruley J. (2015). Template-assisted Selective Epitaxy of III-V Nanoscale Devices for Co-planar Heterogeneous Integration with Si. Appl. Phys. Lett. 106, 233101. 10.1063/1.4921962

[B21] Shi C. Dong Y. Li Q. (2019). High-Performance Nonequilibrium InSb PIN Infrared Photodetectors. IEEE Trans. Electron. Devices 66, 1361–1367. 10.1109/TED.2019.2895032

[B22] Werner K. Beyer A. Oelerich J. O. Baranovskii S. D. Stolz W. Volz K. (2014). Structural Characteristics of Gallium Metal Deposited on Si (001) by MOCVD. J. Cryst. Growth 405, 102–109. 10.1016/j.jcrysgro.2014.07.045

[B23] Xie C. Pusino V. Khalid A. Aziz M. Steer M. J. Cumming D. R. S. (2016). “Monolithic Fabrication of InSb-Based Photo-Pixel for Mid-IR Imaging,” in 2016 Compound Semiconductor Week (CSW) [Includes 28th International Conference on Indium Phosphide & Related Materials (IPRM) & 43rd International Symposium on Compound Semiconductors (ISCS), Toyama, Japan, 26–30.06.2016 (IEEE), 1–2. 10.1109/ICIPRM.2016.7528581

